# Head and Neck Cancer Tumor Segmentation Using Support Vector Machine in Dynamic Contrast-Enhanced MRI

**DOI:** 10.1155/2017/8612519

**Published:** 2017-09-07

**Authors:** Wei Deng, Liangping Luo, Xiaoyi Lin, Tianqi Fang, Dexiang Liu, Guo Dan, Hanwei Chen

**Affiliations:** ^1^Department of Radiology, Guangzhou Panyu Central Hospital, Guangzhou, China; ^2^Medical Imaging Institute of Panyu, Guangzhou, China; ^3^Medical Imaging Center, The First Affiliated Hospital of Jinan University, Guangzhou, China; ^4^National-Regional Key Technology Engineering Laboratory for Medical Ultrasound, Guangdong Key Laboratory for Biomedical Measurements and Ultrasound Imaging, School of Biomedical Engineering, Health Science Centre, Shenzhen University, Shenzhen, China; ^5^Center for Neurorehabilitation, Shenzhen Institute of Neuroscience, Shenzhen 518057, China

## Abstract

**Objective:**

We aimed to propose an automatic method based on Support Vector Machine (SVM) and Dynamic Contrast-Enhanced Magnetic Resonance Imaging (DCE-MRI) to segment the tumor lesions of head and neck cancer (HNC).

**Materials and Methods:**

120 DCE-MRI samples were collected. Five curve features and two principal components of the normalized time-intensity curve (TIC) in 80 samples were calculated as the dataset in training three SVM classifiers. The other 40 samples were used as the testing dataset. The area overlap measure (AOM) and the corresponding ratio (CR) and percent match (PM) were calculated to evaluate the segmentation performance. The training and testing procedure was repeated for 10 times, and the average performance was calculated and compared with similar studies.

**Results:**

Our method has achieved higher accuracy compared to the previous results in literature in HNC segmentation. The average AOM with the testing dataset was 0.76 ± 0.08, and the mean CR and PM were 79 ± 9% and 86 ± 8%, respectively.

**Conclusion:**

With improved segmentation performance, our proposed method is of potential in clinical practice for HNC.

## 1. Introduction

Head and neck cancer (HNC) is an aggressive cancer at the head and neck region with high incidence in southern China especially in Hong Kong and Guangdong [1]. Medical imaging has been very important in the diagnosis and treatment of HNC. Dynamic contrast-enhanced magnetic resonance imaging (DCE-MRI) is an imaging method in which T1-weighted MRI scans are acquired dynamically after injection of MRI contrast agent, providing information about the characteristics of the physiological procedure. DCE-MRI tracks the diffusion of the contrast agent (a paramagnetic substance, normally Gadolinium-based) over time into the tissue by repeated imaging to reflect hemodynamic information such as the formation and permeability of microvascular in living tumor [2]. The DCE-MRI image stores the time-intensity curve (TIC), which is different among tissues, like cancer, normal soft tissue, bone, and so on. Compared with the traditional MRI images and CT images, the differences in DCE-MRI images among tissues are more characteristic [3].

The diagnosis and treatment of HNC require accurate tumor lesion segmentation. Regarded as the ground truth, artificial segmentation operated by experienced radiologists is nonetheless time-consuming, and the accuracy is limited by the experience of radiologists. In recent years, automatic segmentation has attracted much attention. Machine learning algorithms have been applied in the segmentation of HNC, such as supervised learning, unsupervised learning, semisupervised learning, and enhanced learning. These automatic segmentation methods may reduce the subjectivity and improve the quality in the segmentation tasks.

Among these methods, Support Vector Machine (SVM), a supervised learning algorithm, has showed great superiority with small sample size of data [4]. In this study we aimed to develop an automatic segmentation method for HNC based on DCE-MRI by using SVM.

## 2. Materials and Methods

### 2.1. DCE-MRI Data

In our study, all subjects were recruited from The First Affiliated Hospital, Sun Yat-sen University. DCE-MRI was performed on a 3.0-T system (Magnetom Trio, Siemens) with field of view (FOV) of 22 × 22 × 6 cm (AP × RL × FH), a flip angle of 15°, and scanning time of 6 minute 47 seconds with 65 dynamic scans, 5.9 seconds per scan. The contrast agent gadodiamide Gd-DTPA (Omniscan; Nycomed, Oslo, Norway) was injected intravenously as a bolus into the blood at around the 8th dynamic acquisition using a power injector system (Spectris; Medrad, Indianola, Pennsylvania), immediately followed by a 25-mL saline flush at a rate of 3.5 mL per second. The dose of Gd-DTPA was 0.1 mmol/(kg body weight) for each patient. The reconstructed DCE-MRI images were a 4D matrix (144 × 144 × 20 × 65) with 20 slices.

One hundred and twenty samples of DCE-MRI images containing the HNC tumor lesions were used as our database. Each sample was the DCE-MRI time series of a slice and thus was a 144 × 144 × 65 matrix. Eighty samples were selected randomly as the training dataset while the remaining 40 samples were the testing dataset to verify the accuracy of segmentation.

### 2.2. Feature Extraction

Before extracting the features from the TIC in the DCE-MRI images, we performed the normalization as [8]

(1) $$\begin{array}{c} i_{real}\left(\;t\;\right)=\frac{i_{n}\left(t\right)-i\left(pre\right)}{i\left(pre\right)}, \end{array}$$

where *i*_real_(*t*) denotes the final normalized TIC, *i*_*n*_(*t*) denotes the original TIC, and *i*(pre) denotes the average intensity in the first eight scans (before the injection of contrast agent) of *i*_*n*_(*t*).

In several studies some features had already been extracted from DCE-MRI images and successfully applied to classify the tumors from the surrounding tissue [8, 9]. In our study, with the normalized TIC (*i*_real_(*t*)), the same TIC features were calculated. The maximum intensity was calculated as

(2) $$\begin{array}{c} i_{max}=\max\left(\;i_{real}\left(\;t\;\right)\;\right). \end{array}$$

The time of reaching the maximum intensity, namely, time to peak, was calculated as

(3) $$\begin{array}{c} i_{real}\left(\;t_{peak}\;\right)=max\left(\;i_{real}\left(\;t\;\right)\;\right). \end{array}$$

The onset time was defined as the time to reach 10% of the maximum signal intensity after the 8th time point:

(4) tonset=t10%−t8,it10%=imax∗10%.

The wash-in rate was defined as the mean gradient between the two time points of *t*_onset_ and the maximum intensity:

(5) $$\begin{array}{c} \Delta_{washin}=\frac{\left(i_{real}\left(t_{peak}\right)-i_{real}\left(t_{onset}\right)\right)}{\left(t_{peak}-t_{onset}\right)}. \end{array}$$

The wash-out rate was defined as the mean gradient between *t*_peak_ and the 65th time point:

(6) $$\begin{array}{c} \Delta_{washout}=\frac{\left(i_{real}\left(t_{peak}\right)-i_{real}\left(t_{65}\right)\right)}{\left(t_{65}-t_{peak}\right)}. \end{array}$$

Besides, we also used Principal Component Analysis (PCA) [10] in this study to extract the principal components of the TIC. We chose the first two components (the eigenvector with the two highest eigenvalues) from PCA results and then multiplied them by the original data to produce two features. These two new features were used in the segmentation tasks.

### 2.3. SVM Training and Testing

#### 2.3.1. SVM Training

For the training dataset of 80 samples, we firstly carefully drew some rectangular regions of interest (ROIs) for 4 regions, namely, the tumor lesions, the vessels, the normal tissue, and the cavity. This was done by an experienced radiologist (Dr. Wei Deng, 12 years' experience in Radiology) in ImageJ (National Institutes of Health, Bethesda, MD) and double-checked by another experienced radiologist with 14 years' experience who were blind to our study. We then calculated the mean TIC curve for the four regions, respectively, in the 80 samples as

(7) $$\begin{array}{c} i_{aver}\left(\;t,j\;\right)=\frac{\sum_{j=1}^{k}i\left(t,j\right)}{k},\;t=1,\ldots ,65, \end{array}$$

where *i*_aver_(*t*, *j*) denotes the mean TIC in this ROI, *i*(*t*, *j*) denotes the TIC of a voxel, and *k* denotes the total number of voxels. Thus, with 80 samples, we obtained 80 × 4 average TICs. We then calculated the 7 features (5 TIC characteristics, and 2 by PCA) for all the 320 TICs. We labeled these features with their corresponding type (tumor, vessel, normal tissue, and cavity). These features and labels formed our training dataset.

For SVM training, we used the MATLAB toolbox libsvm 3.17 (http://www.csie.ntu.edu.tw/~cjlin/libsvm/). After normalized across different samples, the training dataset was used to train three SVM classifiers. We tried and compared between the five curve features and the two PCA features and selected the PCA features in training the SVM classifier for classifying between cavity and the other three tissues, the 5 TIC features in classifying the normal tissue and blood vessels from the other tissues. The radial basis function (RBF) kernel was used in libsvm. The parameters of *C* and *g* in libsvm 3.17 were selected by cross-validation and the grid-search technique.

#### 2.3.2. SVM Testing

Before segmentation, a rectangular ROI was roughly drawn in each of the 40 testing samples. We then applied the three trained classifiers to these ROIs for voxel-by-voxel classification. First, the voxels of vessels were classified by the first classifier. Then the voxels in cavity were also classified by the second classifier. Finally, the voxels in normal tissues were also classified. We removed all the voxels classified above, and thus the tumor lesions were ultimately segmented.

To evaluate the segmentation performance of our method, we compared the automated segmentation results with the ground truth and calculated the area overlap measure (AOM) as

(8) $$\begin{array}{c} AOM=\frac{A_{R}\cap A_{G}}{A_{R}\cup A_{G}}, \end{array}$$

where *A*_*R*_ is the segmentation results and *A*_*G*_ is the ground truth. Again, the ground truth for the tumor lesions in these 40 testing samples was manually drawn by an experienced radiologist (Dr. Wei Deng) and double-checked by another experienced radiologist with 14 years' experience who was blind to our study.

To evaluate the superiority of our proposed method to other studies, the corresponding ratio (CR) and percent match (PM) were also calculated as

(9) CR=TP−0.5×FPGT×100%,PM=TPGT×100%,

where true positive (TP) denotes the correctly identified tumor region, false positive (FP) denotes the tumor lesion that was incorrectly predicted as nontumor tissue, and the ground truth (GT) denotes the correct tumor region drawn by the radiologist. We repeated the above training and testing for 10 times in order to calculate the mean value of AOM, CR, and PM.

## 3. Results

**Figure 1:**
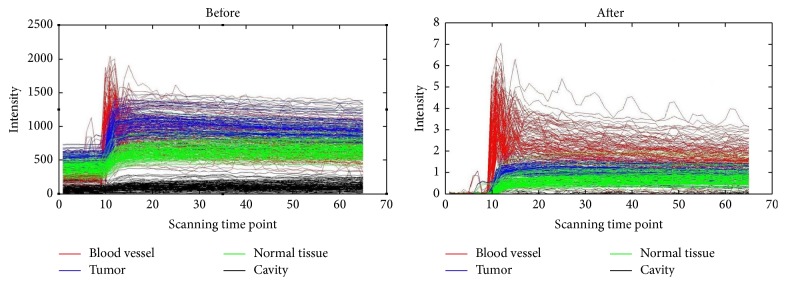
The time-intensity curve (TIC) of four regions before and after normalization.

**Figure 2:**
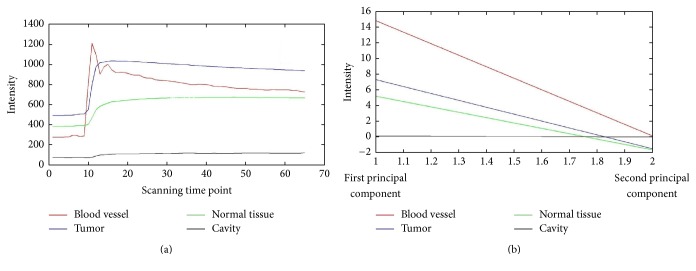
The average original time-intensity curves (TICs) of different subregions in a region of interest (ROI) and the two components extracted by using Principal Component Analysis (PCA). (a) The average TICs of different regions. (b) The two components selected by PCA of the normalized TIC.

The unnormalized and normalized TICs of four different regions of one sample were shown in Figure 1. As shown, after normalization, the TICs of different regions were well distinguished between each other.  Figure 2(a) shows the average original TICs of different regions in a typical training sample, and Figure 2(b) shows the two components selected by PCA.

**Figure 3:**
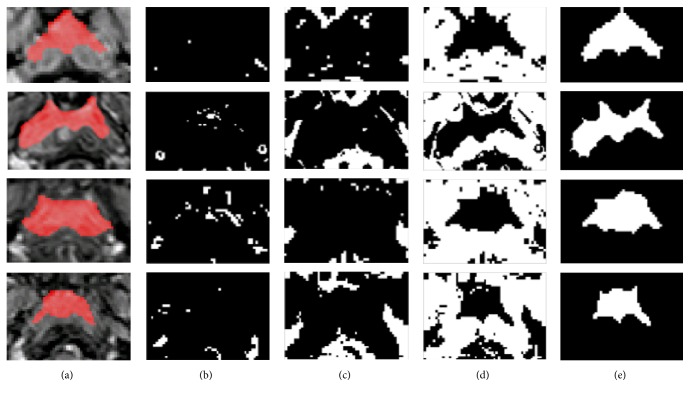
Examples of tumor segmentation results from four patients versus the ground truth. Row-wise, from top to bottom, corresponding to 4 typical samples with AOM of 0.89, 0.79, 0.67, and 0.71, respectively. (a) Ground truth of the tumor drawn by an experienced radiologist (in red); (b) the blood vessels identified by the first classifier (in white); (c) the cavity identified by the second classifier (in white); (d) the normal tissue identified by the third classifier (in white); (e) the tumor region (in white) segmented by removing the voxels identified in (b), (c), and (d) from the region of interest (ROI).

HNC tumor segmentation by using the proposed method was successfully performed on the 40 testing samples. The mean AOM was 0.76 with standard deviation of 0.08.  Figure 3 shows four typical cases of HNC lesion segmentation, including the ground truth in Figure 3(a) and the automated segmentation results in Figures 3(b)–3(e).

The comparison of segmentation performance between our method and the similar studies is summarized in Table 1. By our method, the mean CR was 79 ± 9%, and the mean PM was 86 ± 8%, which were both higher than those in the previous studies.

## 4. Discussion

In this study, a SVM-based method for tumor segmentation in DCE-MRI images of HNC was proposed. Experimental results indicated that this proposed method could effectively segment HNC lesions with high accuracy. We achieved an average AOM of 0.76 ± 0.08. Compared with the SVM-based method proposed in the previous studies [7], the CR value of 79 ± 9% (72 ± 6%) and the PM value 86 ± 8% (79 ± 7%) in our study were both higher. Compared with other methods about HNC tumor segmentation [5–7], our method also showed higher CR and PM values.

There may be several reasons for better performance of our method. Firstly, the normalized TICs makes the data dimensionless and comparable. As shown in Figure 1, before normalization, the TICs of different tissues especially blood vessel and tumor region are similar, while, after normalization, they are well distinguished and meanwhile the differences of TIC are more obvious.

In addition, the extraction and selection of features are essential in segmentation tasks. We chose the features by using PCA and the features of TIC change for the three classifiers. On the one hand, we found that the classification performance of the PCA features in cavity was more obvious. As shown in Figure 2, by PCA, although only two principal components are shown, the differences in curve variation are still obvious and the computational expense is reduced. On the other hand, we believed that the combination of different SVM classifiers with different features improves the accuracy of segmentation. In our method with three SVM classifiers, blood vessel, cavity, and normal tissue have been classified independently and successively (as shown in Figure 3). As a supervised learning algorithm, SVM has shown a strong learning ability [4]; thus, with more training samples, the classification performance can be better.

Our study has several limitations. In fact, there is a thin layer of mucosa membrane around the HNC tumor. This tissue might be an obstacle while designing the algorithm based on TIC features, because the TIC is quite similar to the HNC tumor. In the future, we intend to incorporate the high-resolution MRI images for better classification between these two. Another way to improve our method may be the deep learning-based approaches, with which we may obtain more discriminative features and yield improved performance [11].

## 5. Conclusion

We successfully proposed an automatic segmentation method based on SVM for HNC. The results of this study showed that the segmentation performance was superior to previous studies. Our method, if was further verified with more data, is of potential in the clinical practice of HNC patient management.

## Figures and Tables

**Table 1:** Comparisons of PM and CR between the segmentation performance obtained by the proposed SVM method and other methods in literature.

Studies	Algorithm	CR^a^	PM^b^
Huang et al. [5]	HMRF^c^	0.72	0.85
Ritthipravat et al. [6]	Probabilistic Function	0.52	0.85
Zhou et al. [7]	SVM^d^	0.72 ± 0.06	0.79 ± 0.07
Our proposed method	SVM	0.79 ± 0.09	0.86 ± 0.08

^a^Corresponding ratio. ^b^Percent match. ^c^Hidden Markov random field. ^d^Support vector machine.
